# Valorization of acid-hydrolyzed tea stem waste for sustainable biodiesel production using *Chlorella vulgaris*: a biorefinery approach

**DOI:** 10.3389/fbioe.2026.1816002

**Published:** 2026-05-18

**Authors:** Charith Akalanka Dodangodage, R. H. N. S. Rathnapriya, Geethaka Nethsara Gamage, Jagath C. Kasturiarachchi, Thilini A. Perera, Sanjitha Dilan Rajapakshe, Sayuri S. Niyangoda, Rangika Umesh Halwatura

**Affiliations:** 1 ProGreen Lab, Department of Civil Engineering, University of Moratuwa, Moratuwa, Sri Lanka; 2 Department of Agricultural Engineering and Environmental Technology, University of Ruhuna, Matara, Sri Lanka; 3 Department of Applied Sciences, Sri Lanka Institute of Information Technology, Malabe, Sri Lanka; 4 Department of Plant Sciences, University of Colombo, Colombo, Sri Lanka; 5 Department of Biosystems Technology, Uva Wellassa University, Badulla, Sri Lanka; 6 Department of Chemistry, University of Kansas, Lawrence, KS, United States

**Keywords:** acid hydrolysis, Chlorella vulgaris, circular biorefinery, microalgal biodiesel, mixotrophic cultivation, tea stem waste

## Abstract

The prohibitive cost of synthetic cultivation media remains a fundamental bottleneck in the commercial deployment of microalgal biodiesel. This study investigates the valorization of recalcitrant tea stem waste, an abundant agro-industrial by-product, as a low-cost, nutrient-rich medium for *Chlorella vulgaris* within an integrated biorefinery framework. Following thermochemical acid hydrolysis, a two-stage optimization of hydrolysate concentration and incident irradiance was conducted to maximize biomass production. Undiluted (100%) hydrolysate under elevated irradiance (240 µmol photons m^-2^ s^-1^) compensated for optical attenuation in the dark medium and yielded a maximum biomass concentration of 1.65 ± 0.07 g L^-1^, representing an approximately 5-fold increase over the synthetic Bold’s Basal Medium (BBM) control. Concurrently, substantial nutrient recovery was achieved, with 83.23% nitrate and 95.60% phosphate assimilation by Day 10. The resulting nutrient limitation acted as a secondary abiotic stressor, triggering enhanced intracellular lipid accumulation and yielding a peak volumetric lipid concentration of 0.094 ± 0.005 g L^-1^, approximately 4.5-fold higher than the autotrophic control. Fatty acid methyl ester (FAME) profiling revealed a saturated-dominant composition (85.57% SFA), corresponding to favorable predicted biodiesel properties, including low iodine value and high cetane number, consistent with international fuel standards. Overall, this study establishes tea stem hydrolysate as an efficient integrated cultivation matrix for simultaneous mixotrophic growth and lipid biosynthesis, advancing a scalable circular waste-to-energy pathway for agro-industrial systems.

## 1 Introduction

The continued rise in global energy demand is intensifying concerns over energy security and climate impacts. In 2024, globalenergy demand increased by 3.4%, driven largely by rapid growth in electricity use, and energy-related CO_2_ emissions increased by 0.8% to a record 37.8 Gt CO_2_ (Nassar and Khaleel, 2024; Energy Agency, 2025). This trajectory reinforces the need for scalable low-carbon fuel pathways that can complement power-sector decarbonization, particularly for heavy-duty and aviation applications where direct electrification remains constrained by energy density limitations. Consequently, there is an accelerating transition toward third-generation microalgal feedstocks, which can sustainably decouple renewable fuel production from agricultural land and freshwater reliance within circular bioeconomy frameworks (Tomar et al., 2023).

Within the portfolio of renewable liquid fuels, microalgae-derived biodiesel remains of interest due to the potential for high areal productivity and cultivation on non-arable land using non-conventional nutrient sources (Casanova et al., 2023; Zhang et al., 2022; Kanwal et al., 2025). Among candidate strains, *Chlorella vulgaris* is widely investigated owing to its physiological robustness, rapid growth, and capacity to accumulate intracellular lipids under appropriate cultivation and stress conditions. Furthermore, its well-documented high tolerance to complex organic matrices and phenolic compounds makes it uniquely suited for mixotrophic cultivation in waste-derived effluents. However, despite extensive laboratory-scale demonstrations, the translation of microalgal biodiesel into industrial practice remains limited by upstream production costs (Mallick et al., 2012; Liu et al., 2025; khalaji, 2022).

A dominant contributor to operating expenditure is the cultivation medium. Conventional formulations rely on refined sources of nitrogen, phosphorus, and trace minerals, which can account for up to 50%–70% of total biomass production costs. Consequently, replacing synthetic nutrients with low-cost, waste-derived reservoirs has emerged as a key strategy to improve techno-economic feasibility (Kadier et al., 2021; Ullah et al., 2022; Dodangodage et al., 2026a). Within a circular bioeconomy framework, utilizing agro-industrial residues not only lowers nutrient procurement costs but also provides distinct metabolic advantages (Ezhumalai et al., 2024; Zabochnicka et al., 2022). Under mixotrophic conditions, microalgae simultaneously utilize light and organic carbon, which can enhance growth rates and influence lipid accumulation. However, the success of this approach depends on selecting an abundant, non-food-competitive waste stream capable of providing bioavailable nutrients and carbon without generating strong inhibitory effects (Wu et al., 2025; Veronesi et al., 2020; Gao et al., 2019; Dodangodage et al., 2026b).

Among globally available residues, tea industry by-products represent a substantial and underutilized lignocellulosic resource with documented potential for valorization. Tea wastes contain structurally bound carbohydrates (cellulose and hemicellulose), lignin, proteins, minerals, and bioactive compounds such as polyphenols and tannins (Çakmak et al., 2024; Xu et al., 2023). However, it is important to distinguish tea stem waste from general tea leaf residues. Stems exhibit a highly polymerized lignocellulosic architecture, specifically regarding lignin and crystalline cellulose, that restricts nutrient mobilization and reduces the immediate availability of fermentable carbon and soluble nitrogen in the liquid phase (Çakmak et al., 2024; Zhang et al., 2024).

Accordingly, valorization of tea stems as a cultivation substrate requires pretreatment to disrupt the lignocellulosic matrix and release bioavailable constituents. Acid hydrolysis is particularly relevant because it can depolymerize hemicellulosic fractions and facilitate the solubilization of sugars and minerals into a hydrolysate suitable for microalgal mixotrophic assimilation (Porninta et al., 2023; Wang et al., 2025a; Chen et al., 2013; Wang et al., 2025b). At the same time, hydrolysis severity must be carefully controlled. Severe parameters (e.g., high acid concentrations and elevated temperatures) trigger the dehydration of pentose and hexose sugars into inhibitory furanic compounds, such as furfural and hydroxymethylfurfural (Istasse and Richel, 2020). Furthermore, severe hydrolysis conditions increase medium color intensity and turbidity. This leads to severe light attenuation, which can reduce photosynthetic productivity and overall process performance.

In addition, tea-derived secondary metabolites introduce further process complexity. Phenolic compounds can inhibit microalgal growth at elevated concentrations, while moderate concentrations may be tolerated and can influence stress responses and carbon partitioning (Zheng et al., 2023; Ni et al., 2023). Therefore, the success of tea stem hydrolysate as a cultivation medium depends not only on nutrient release, but also on balancing inhibitor formation and optical properties to maintain favorable growth conditions.

From a biodiesel perspective, productivity must be evaluated jointly with lipid content and fatty acid composition, as these determine the quality of fatty acid methyl esters (FAME) and their predicted fuel properties. In many oleaginous microalgae, nitrogen limitation is a reliable trigger that redirects carbon flux away from protein synthesis and toward neutral lipid storage (triacylglycerols, TAG) (Palanisamy et al., 2023; Adams et al., 2013; Liu et al., 2016; Bharti et al., 2024). This metabolic shift can alter the degree of fatty acid saturation and thereby influence key biodiesel-relevant properties. Consequently, a tea stem hydrolysate that supports rapid early growth but enables controlled onset of nutrient limitation could provide a practical route to enhance lipid productivity within an integrated waste-to-fuel biorefinery framework (Brandenburg et al., 2018; Farouk et al., 2024).

Despite the relevance of tea wastes for circular bioeconomy applications, key knowledge gaps remain. While prior studies have successfully utilized general tea leaf waste or fermented tea extracts for microbial growth, systematic evidence targeting the highly lignified tea stem fraction as a primary nutrient source remains scarce. Furthermore, the relationship between acid-hydrolysis severity and (i) nutrient solubilization, (ii) formation of inhibitory compounds, and (iii) hydrolysate optical properties has not been sufficiently resolved for reliable cultivation design. Additionally, dark and turbid hydrolysates may impose light limitation, yet quantitative assessments linking hydrolysate loading to growth kinetics and lipid accumulation across irradiance levels remain underreported. Finally, the downstream implications for FAME profiles and predicted biodiesel quality when using tea stem hydrolysates remain insufficiently characterized.

Based on these gaps, we hypothesized that the recalcitrant lignocellulosic matrix of tea stem waste can be selectively depolymerized to generate a nutrient-rich hydrolysate capable of sustaining mixotrophic growth of *C. vulgaris* without severe inhibitory effects. Accordingly, this study aims to develop an integrated bioprocess for the valorization of acid-hydrolyzed tea stem waste for sustainable biodiesel production. Specifically, (i) hydrolysis conditions were optimized to maximize nutrient solubilization while minimizing inhibitor formation, (ii) the resulting hydrolysate was evaluated across a range of loading concentrations and photon flux densities to identify optimal growth conditions under light-limited environments, and (iii) biomass composition and fatty acid profiles were analyzed to assess biodiesel suitability. This study establishes a scalable circular biorefinery framework that integrates lignocellulosic waste valorization with microalgal biofuel production.

## 2 Materials and methods

### 2.1 Tea stem waste collection and pre-treatment

Tea stem waste (*Camellia sinensis*) was collected directly from the Mathugama Tea Factory, an industrial tea processing facility located in Mathugama, Kalutara District, Sri Lanka (6 °32′27.8″N, 80 °06′36.0″E). To provide a baseline understanding of the substrate prior to thermochemical disruption, it is well-documented in the literature that raw *C. sinensis* stems are highly lignified. While the exact elemental composition of this specific batch was not quantified prior to pretreatment, standard profiles for raw tea stems indicate a recalcitrant matrix characterized by high structural carbon and extremely low soluble nitrogen and phosphorus. This inherent lack of bioavailable nutrients underscores the necessity of the subsequent acid hydrolysis step to mobilize fermentable sugars and essential minerals for microalgal assimilation (Porninta et al., 2023; Wang et al., 2025a). To minimize biological degradation and maintain the representativeness of the industrial by-product stream, samples were collected into sterile polyethylene containers, transported at ambient conditions, and processed within 24 h. Upon arrival, the stems were washed thoroughly with distilled water to remove adhering dust and extraneous particulates, then oven-dried at 60 °C until a constant mass was obtained. The dried stems were mechanically milled using a laboratory blender and sieved to obtain a uniform powder (particle size <1 mm), thereby increasing accessible surface area to support thermochemical hydrolysis.

### 2.2 Acid hydrolysis and preparation of tea stem hydrolysate medium

Thermochemical acid hydrolysis was used to depolymerize the lignocellulosic matrix and mobilize soluble nutrients and organic carbon into the aqueous phase. Tea stem powder was mixed with 3% (v/v) sulfuric acid (H_2_SO_4_) at a solid-to-liquid ratio of 1:8 (w/v) (9 g in 72 mL) and subjected to autoclave-assisted hydrolysis at 121 °C for 25 min. After hydrolysis, the slurry was cooled to room temperature and filtered through sterile muslin cloth to separate residual solids. The clarified filtrate was diluted with distilled water to a final working volume of 1.5 L to obtain the 100% (v/v) tea stem hydrolysate stock medium. The pH was adjusted to 6.8 ± 0.1 using 1 M NaOH. Standard Bold’s Basal Medium (BBM) was prepared as the synthetic control using analytical grade chemicals purchased from (Sigma-Aldrich, St. Louis, Missouri, United States). The BBM composition consisted of (per liter): 250 mg NaNO_3_, 25 mg CaCl_2_.2H_2_O, 75 mg MgSO_4_.7H_2_O, 75 mg K_2_HPO_4_, 175 mg KH_2_PO_4_, 25 mg NaCl, 50 mg EDTA, 31 mg KOH, 4.98 mg FeSO_4_.7H_2_O, and 1 mL of trace element solution (11.42 mg H_3_BO_3_, 8.82 mg ZnSO_4_.7H_2_O, 1.44 mg MnCl_2_.4H_2_O, 0.71 mg MoO_3_, 1.57 mg CuSO_4_.5H_2_O, and 0.49 mg Co.(NO_3_)_2_·6H_2_O) (Bischoff, 1963). All media (BBM and hydrolysate-based media) were then sterilized by autoclaving at 121 °C for 20 min and cooled to room temperature prior to inoculation.

Initial physicochemical characterization of the hydrolysis was conducted in accordance with APHA standard methods. All measurements were performed in triplicate (*n* = 3). The following parameters were analyzed (Association, 1926).

- pH–Measured using a benchtop pH meter (SevenCompact, Mettler Toledo, Switzerland).
- Nitrate (NO_3_^−^–N) – UV absorbance at 220 nm with baseline correction at 275 nm using a UV–Vis spectrophotometer (UV-1800, Shimadzu Corp., Kyoto, Japan).
- Phosphate (PO_4_^3-^–P) – molybdenum blue method, absorbance at 880 nm.

### 2.3 Microalgal strain and inoculum preparation

An axenic culture of *C. vulgaris* (isolated and maintained locally) was obtained from the ProGreen Laboratory, University of Moratuwa, Sri Lanka. Strain integrity and culture purity were monitored by regular microscopic observation. Pre-cultures were maintained in BBM at 28 °C ± 2 °C under continuous illumination (140 µmol photons m^-2^ s^-1^; cool-white LEDs) with continuous aeration (0.5 vvm; sterile-filtered air). Cells were harvested during late exponential phase by centrifugation (4,500 × *g*, 20 min) and immediately used to inoculate experimental reactors to reduce adaptation lag (Dodangodage et al., 2026c).

Cultivation experiments were performed in 2 L laboratory-scale glass photobioreactors with a working volume of 1.8 L. Each reactor was fitted with a three-port GL45 screw cap to enable aseptic aeration, sampling, and pressure relief. Aeration lines were equipped with 0.45 µm PTFE membrane filters to maintain sterility. Experimental cultivations were operated under a 12:12 h light–dark photoperiod provided by symmetrically positioned broad-spectrum white light-emitting diode (LED) strips (correlated color temperature of approximately 6500 K) (Dodangodage et al., 2026c; Naseema Rasheed et al., 2023; Mattos et al., 2012; Nicodemou et al., 2024).

### 2.4 Experimental design and cultivation conditions

#### 2.4.1 Phase I: screening of hydrolysate dilution ratio

**FIGURE 1:**
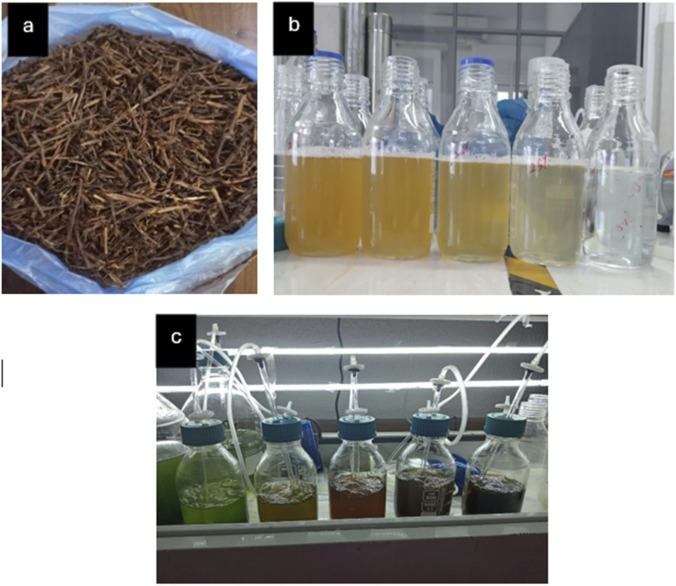
Representative images of **(a)** raw tea stem waste, **(b)** hydrolysate at different dilution levels, and **(c)** microalgal cultivation setup 2.4.2. Phase II: Main Cultivation.

A concentration screening was conducted to evaluate nutrient loading and potential substrate inhibition. Four hydrolysate dilutions (25%, 50%, 75%, and 100% v/v) were prepared from the sterilized 100% stock using sterile distilled water (Figure 1). BBM was used as the synthetic control. Cultures were maintained at 28 °C ± 2 °C, aerated continuously (0.5 vvm), and illuminated at 140 µmol photons m^-2^ s^-1^ under a 12:12 h photoperiod. Growth was monitored daily by optical density and every 48 h by gravimetric dry cell weight (Section 2.5.1).

#### 2.4.2 Phase II: Optimization of light intensity

Based on Phase I, the 100% hydrolysate medium was selected for irradiance optimization to evaluate growth performance under potential light-attenuation associated with the dark hydrolysate. Cultures were exposed to four photon flux densities (60, 120, 180, and 240 µmol photons m^-2^ s^-1^), while all other conditions were maintained as described in Phase I. Light intensity incident at the reactor surface was measured using a calibrated quantum sensor prior to each run and periodically checked throughout cultivation.

#### 2.4.3 Phase III: main cultivation

**FIGURE 2:**
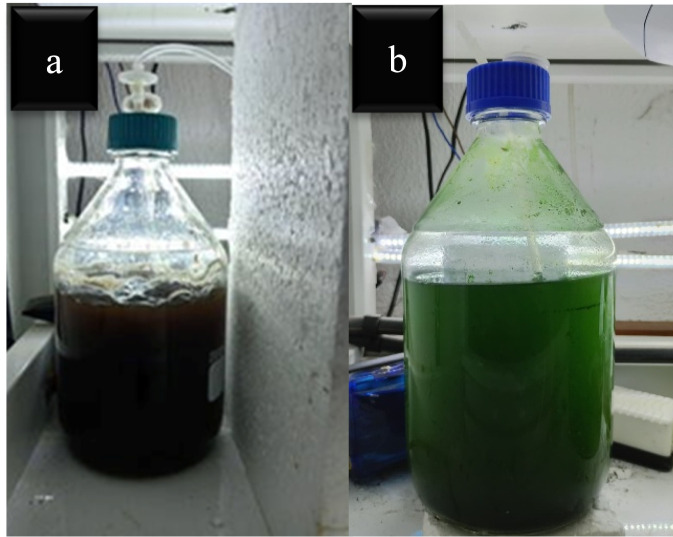
Inoculated *Chlorella vulgaris* in **(a)** tea stem waste hydrolysate and **(b)** BBM (control) media.

The main batch cultivation was performed using 100% tea stem hydrolysate under the optimized irradiance (240 µmol photons m^-2^ s^-1^). In contrast, the BBM control was maintained at a baseline irradiance of 140 µmol photons m^-2^ s^-1^. This elevated light intensity for the hydrolysate was specifically selected to compensate for severe light attenuation caused by the inherent dark color and turbidity of the medium, ensuring adequate photon penetration to support baseline photosynthetic activity in the dense matrix. A 12-day cultivation period was used to capture exponential growth and subsequent lipid accumulation during stationary phase. Biomass accumulation and nutrient depletion dynamics were quantified throughout the cultivation cycle (Figure 2).

### 2.5 Analytical procedures

#### 2.5.1 Biomass concentration

Biomass was monitored at 48 h intervals. Optical density was recorded at 680 nm (OD_680_) using a UV–Vis spectrophotometer (Shimadzu UV-1800, Japan). To account for the inherent turbidity and background absorbance of the hydrolysate, all OD_680_ measurements were strictly blanked against an uninoculated control of the respective cultivation medium. Dry cell weight (DCW) was determined gravimetrically: 5 mL aliquots were vacuum-filtered through pre-dried, pre-weighed glass microfiber filters (GF/C, 1.2 μm; Hyundai Micro, South Korea), then oven-dried at 60 °C for 24 h to constant mass. DCW was calculated using (Dodangodage et al., 2026a; Dodangodage et al., 2026c):

$$\text{Dry Weight }\left(g\;L^{-1}\right)\;\left(DW_{n}\right)=\frac{W_{2}-W_{1}}{v}$$

where $W_{2}$ is the final filter mass with biomass (g), $W_{1}$ is the initial filter mass (g), and V is the filtered culture volume (L). OD_680_ was used as a rapid growth proxy, while DCW served as the quantitative biomass metric.

#### 2.5.2 Nutrient and COD removal

To evaluate resource recovery and organic load reduction during cultivation, nitrate, phosphate, COD, and pH were measured at 2-day intervals. Samples (5 mL) were centrifuged (10,000 × g, 5 min) and the supernatant was filtered through 0.22 μm syringe filters to obtain the dissolved fraction for analysis. pH was measured using a calibrated multi-parameter probe (Hanna HI98194). Removal efficiency (RE) was computed using (Dodangodage et al., 2026a):

Removal efficiency (RE, %) was calculated as:

$$\left(\text{RE}\right)=\frac{c_{i}‐c_{f}}{c_{i}}\times 100$$

where $C_{i}$ and $C_{f}$ are the initial and final concentrations (mg L^-1^), respectively.

#### 2.5.3 Analytical quality control

All analyses were performed using biological triplicates with technical replication where applicable. Instrument blanks and reagent controls were included with each batch. Calibration curves were prepared using appropriate analytical-grade standards and exhibited linearity of $R^{2}\geq 0.996$ .

### 2.6 Biomass harvesting and lipid extraction

At the end of the 12-day batch period, biomass was harvested by centrifugation (4,500 x *g*, 10 min; Eppendorf 5810R, Germany), washed twice with distilled water, and oven-dried at 60 °C for 24 h to constant mass. Total lipids were extracted using a modified Bligh and Dyer method (Bligh and Justin Dyer, 1959). Briefly, dried biomass was homogenized in chloroform:methanol (1:1, v/v). Phase separation was induced using 0.8% (w/v) NaCl to achieve a final chloroform:methanol:NaCl ratio of 10:10:9 (v/v/v). The lower chloroform phase was recovered and evaporated under nitrogen. Residual solvent was removed at 60 °C to constant mass, and lipid content was expressed as wt% of dry biomass (Lee et al., 2013; Dodangodage et al., 2025a; Akondi et al., 2017; Harrison et al., 2022).

### 2.7 FAME preparation and fatty acid profiling

Extracted lipids were dissolved in chloroform and supplemented with heptadecanoic acid (C17:0) as an internal standard prior to solvent evaporation. Transesterification was performed using 1% (v/v) H_2_SO_4_ in methanol (5 mL), incubated overnight at 50 °C. FAMEs were extracted into *n*-hexane and analyzed using GC–FID (7890A, Agilent, United States of America) with a DB-23 column (60 m × 0.25 mm × 0.15 μm). Injector temperature was 250 °C (1 μL injection; split 1:50). Helium carrier gas was maintained at 230 kPa. The oven program was: 50 °C (1 min), ramp 25 °C min^-1^–175 °C, then 4 °C min^-1^–230 °C (5 min hold). Detector temperature was 280 °C with hydrogen/air/helium flows of 40/450/30 mL min^-1^. Fatty acids were identified and quantified using a 37-component FAME standard mixture (Breuer et al., 2013; Ma et al., 2016).

### 2.8 Biodiesel property prediction based on fatty acid composition

The physicochemical properties of biodiesel derived from microalgal lipids were predicted theoretically based on the fatty acid methyl ester profile using established empirical correlations (Islam et al., 2013; Mondal et al., 2021). The formulas utilized to determine these properties incorporate the following defined variables: (i) represents the specific fatty acid component, A_i_ is the mass percentage of the specific fatty acid, MW_i_ is the molecular weight of the component, and D represents the number of double bonds in that specific fatty acid.

The Iodine Value (IV) was calculated as:

$$IV=\sum \frac{254\times D\times A_{i}}{MW_{i}}$$

The Kinematic Viscosity (ln ν) was determined using the logarithmic mixing rule to account for non-linear blending interactions between fatty acid esters:

$$\ln\nu_{mix}=\sum A_{i}\ln\nu_{i}$$

where νᵢ represents the kinematic viscosity of the individual fatty acid methyl ester component.

The Saponification Value (SV) was calculated as:

$$SV=\sum \frac{560\times A_{i}}{MW_{i}}$$

The Higher Heating Value (HHV) was estimated as:

$$HHV=49.43-\left(0.041\times SV\right)-\left(0.015\times IV\right)$$

The Density (ρ) of biodiesel was calculated using:

$$\rho =\sum A_{i}\rho_{i}$$

where $\rho_{i}=0.8463-\left(0.49/MW_{i}\right)+0.0118\times D$ .

The Cetane Number (CN) was estimated using:

$$\text{CN}=46.3+\left(\frac{5458}{sv}\right)-\left(0.225\times IV\right)OS\left(h\right)=117.9295/\left[\left(XC18:2+XC18:3\right)+2.5905\right]$$

where XC18:2 and XC18:3 correspond to the weight content of linoleic and α-linolenic acids, respectively.

### 2.9 Statistical analysis

All experiments were conducted in independent biological triplicates (*n* = 3). One-way ANOVA was applied for treatment comparisons (hydrolysate concentration screening and light intensity optimization). When significant effects were observed (*p* < 0.05), Tukey’s HSD test was used to distinguish between group means. Both statistical analysis and data visualization were performed entirely using Microsoft Excel (Office 365). Throughout all experimental trials, high reproducibility was achieved, and the standard deviation (SD) was strictly maintained within a narrow range of 2%–5%.

## 3 Results

### 3.1 Physicochemical characterization of tea stem waste hydrolysate

The physicochemical profile of the acid-hydrolyzed tea stem waste utilized in this study is summarized in Table 1. Following thermochemical pretreatment and subsequent neutralization, the hydrolysate pH was established at 6.80 ± 0.10, which falls within a suitable operational range for *Chlorella vulgaris* cultivation. Visually, the medium exhibited a dark-brown, turbid appearance. Such optical characteristics are typical of lignocellulosic hydrolysates and are commonly associated with solubilized aromatic constituents and fine suspended organic particulates generated during hydrolysis and filtration.

**TABLE 1:** Physicochemical characterization of tea stem waste hydrolysate used in the study (*n* = 3).

Parameter	Unit	Value (mean ± SD)
pH (after adjustment)	–	6.80 ± 0.10
Nitrate (NO_3_^−^–N)	mg L^-1^	122.10 ± 4.00
Phosphate (PO_4_^3-^–P)	mg L^-1^	14.90 ± 0.30
Molar N:P ratio (NO_3_^−^–N: PO_4_^3-^–P)	–	12.56: 1
Appearance	–	Dark brown, turbid

Quantitative nutrient profiling confirmed that the applied acid hydrolysis protocol effectively mobilized macronutrients into the aqueous phase. The hydrolysate contained 122.10 ± 4.00 mg L^-1^ nitrate-nitrogen (NO_3_^−^–N) and 14.90 ± 0.30 mg L^-1^ phosphate-phosphorus (PO_4_^3-^–P). The resulting M N:P ratio of 12.56:1 indicates a phosphorus-sufficient (or comparatively nitrogen-limited) nutrient stoichiometry relative to the canonical Redfield ratio (16:1). While absolute nutrient limitation ultimately depends on strain-specific uptake kinetics and cultivation conditions, this baseline profile confirms that the hydrolysate provides a substantial inorganic nutrient reservoir capable of functioning as a standalone cultivation medium.

Overall, the liberated nitrogen and phosphorus concentrations indicate that tea stem waste hydrolysate can support sustained microalgal growth. However, the pronounced color and turbidity of the matrix suggest that optical attenuation, rather than immediate nutrient scarcity, may emerge as a dominant constraint during early-stage cultivation by reducing effective photon availability within the reactor. This physical characteristic provides a direct rationale for the subsequent optimization of incident light intensity to maintain photosynthetic performance under hydrolysate-based cultivation.

### 3.2 Effect of hydrolysate concentration on microalgal growth (screening phase I)

The growth performance of *Chlorella vulgaris* was evaluated in tea stem hydrolysate diluted to 25%, 50%, 75%, and 100% (v/v) and compared against a standard BBM control over a 10-day batch screening period. One-way ANOVA indicated highly significant treatment effects on growth based on both optical density and dry cell weight (*p* < 0.001). Subsequent *post hoc* comparisons (Tukey’s HSD, *p* < 0.05) confirmed that biomass formation differed significantly among the hydrolysate concentrations and relative to the synthetic control.

**FIGURE 3:**
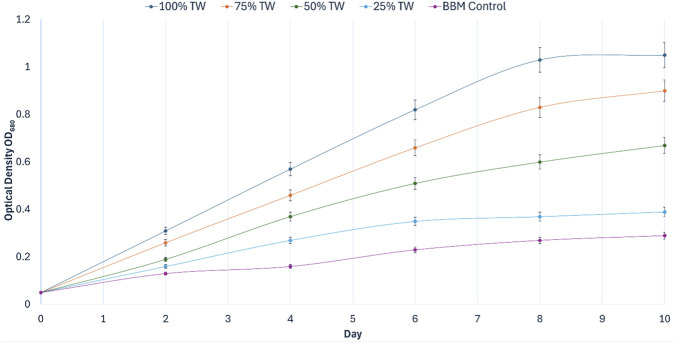
Optical density profiles $\left(OD_{680}\right)$ of *Chlorella vulgaris* cultivated in tea stem hydrolysate at different concentrations (25%, 50%, 75%, and 100% v/v) compared with BBM control during Phase I concentration screening (10-day batch). Values are mean ± SD (*n* = 3).

As shown in Figure 3, *C. vulgaris* exhibited a clear, concentration-dependent increase in optical density across the hydrolysate treatments. Across the tested range, no reduction in growth was observed at higher hydrolysate loadings. All cultures initiated growth with a minimal lag phase; however, divergence among treatments became pronounced by Day 4. The 100% hydrolysate condition supported the fastest early proliferation, reaching an OD_680_ of 0.57 ± 0.02 b y Day 4, compared to 0.16 ± 0.01 in the BBM control. By Day 10, the 100% hydrolysate achieved the highest overall optical density (1.05 ± 0.04), which was 3.6-fold higher than BBM (0.29 ± 0.01) (Tukey’s HSD, *p* < 0.05). The 75% dilution also supported sustained growth (Day 10: 0.90 ± 0.03) but remained statistically inferior to the undiluted hydrolysate.

**FIGURE 4:**
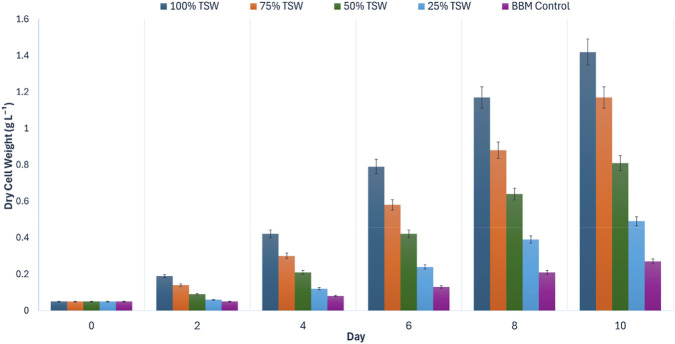
Dry cell weight (DCW, g L^-1^) of *Chlorella vulgaris* cultivated in tea stem hydrolysate at different concentrations (25%, 50%, 75%, and 100% v/v) compared with BBM control during Phase I concentration screening (10-day batch). Values are mean ± SD (*n* = 3).

Gravimetric dry cell weight (DCW) measurements corroborated the optical density trends (Figure 4). The 100% hydrolysate produced the highest final biomass, reaching 1.42 ± 0.06 g L^-1^ on Day 10, whereas the BBM control yielded 0.27 ± 0.01 g L^-1^. Thus, the undiluted hydrolysate generated approximately 5.3-fold higher biomass than the synthetic control. Collectively, these results demonstrate that tea stem hydrolysate provides a bioavailable nutrient matrix capable of supporting significant biomass production across the tested range, with maximum productivity obtained under undiluted conditions.

Based on this superior growth performance, and to maximize industrial waste valorization by minimizing the need for additional freshwater dilution beyond the initial processing volume, the 100% tea stem hydrolysate was selected as the optimal substrate concentration for subsequent irradiance optimization (Phase II).

### 3.3 Optimization of light intensity (screening phase II)

Following the identification of the 100% tea stem hydrolysate as the optimal substrate concentration (Section 3.2), the effect of incident irradiance was evaluated to mitigate optical attenuation associated with the dark, turbid hydrolysate matrix. Cultures were operated across a gradient of photosynthetic photon flux densities (PPFD) at 60, 120, 180, and 240 µmol photons m^-2^ s^-1^ under otherwise identical conditions. A BBM control, maintained under baseline illumination (140 µmol photons m^-2^ s^-1^), was used as a comparative benchmark.

**FIGURE 5:**
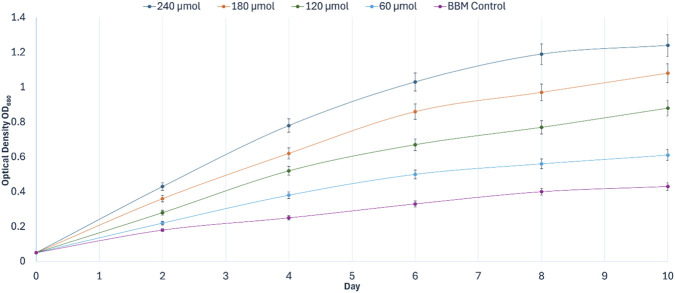
Effect of incident irradiance (PPFD = 60, 120, 180, and 240 µmol photons m^-2^ s^-1^) on $\left(OD_{680}\right)$ of *Chlorella vulgaris* cultivated in 100% tea stem hydrolysate during Phase II light optimization (10-day batch). BBM control was cultivated under baseline illumination (PPFD = 140 µmol photons m^-2^ s^-1^). Values are mean ± SD (*n* = 3).

One-way ANOVA confirmed that irradiance exerted a highly significant effect on biomass accumulation (*p* < 0.001). Post-hoc analysis (Tukey’s HSD, *p* < 0.05) indicated significant differences in growth among the tested PPFD levels. As shown in Figure 5, optical density increased proportionally with increasing PPFD, indicating that photon availability was a dominant constraint on culture performance under hydrolysate-based cultivation.

The highest irradiance condition (240 µmol photons m^-2^ s^-1^) supported the most rapid exponential proliferation and achieved the maximum final optical density (OD_680_ = 1.24 ± 0.06 on Day 10). This value was significantly higher than that obtained at 60 µmol photons m^-2^ s^-1^ (0.61 ± 0.04) and the BBM control evaluated under baseline conditions (0.43 ± 0.02) (Tukey’s HSD, *p* < 0.05). Across the evaluated gradient, no reduction in growth was observed at the highest PPFD, indicating no evidence of photoinhibition within the tested irradiance range, consistent with reduced effective irradiance penetrating the turbid medium.

**FIGURE 6:**
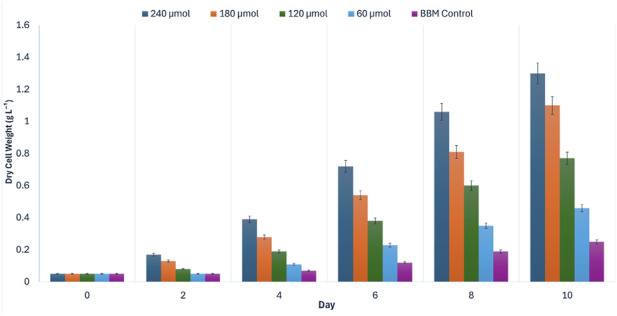
Effect of incident irradiance (PPFD = 60, 120, 180, and 240 µmol photons m^-2^ s^-1^) on dry cell weight (DCW, g L^-1^) of *Chlorella vulgaris* cultivated in 100% tea stem hydrolysate during Phase II light optimization (10-day batch). BBM control was cultivated under baseline illumination (PPFD = 140 µmol photons m^-2^ s^-1^). Values are mean ± SD (*n* = 3).

Gravimetric biomass measurements corroborated the spectrophotometric trends (Figure 6). The maximum dry cell weight (DCW) was recorded at 240 µmol photons m^-2^ s^-1^, reaching 1.30 ± 0.03 g L^-1^ on Day 10. This corresponded to 5.2-fold higher biomass than the respective BBM control (0.25 ± 0.01 g L^-1^) and substantially exceeded the yield obtained at 60 µmol photons m^-2^ s^-1^ (0.46 ± 0.02 g L^-1^) (Tukey’s HSD, *p* < 0.05).

Collectively, these results demonstrate that increasing incident PPFD substantially mitigates light-shading limitations in tea stem hydrolysate and enhances biomass formation. Based on the highest OD_680_ and DCW achieved without detectable growth suppression at elevated irradiance, 240 µmol photons m^-2^ s^-1^ was selected as the optimal illumination level for the subsequent main cultivation experiment (Phase III).

### 3.4 Nutrient uptake and pH evolution during main cultivation

**FIGURE 7:**
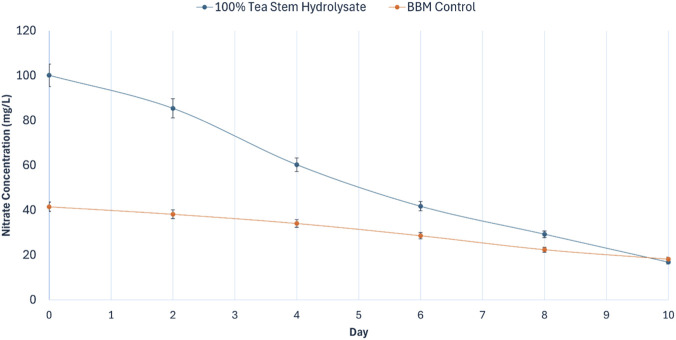
Temporal variation of nitrate-nitrogen (NO_3_^−^–N, mg L^-1^) during main cultivation of *Chlorella vulgaris* in optimized tea stem hydrolysate (100% v/v, PPFD = 240 µmol photons m^-2^ s^-1^) compared with BBM control (PPFD = 140 µmol photons m^-2^ s^-1^). Values are mean ± SD (*n* = 3).

**FIGURE 8:**
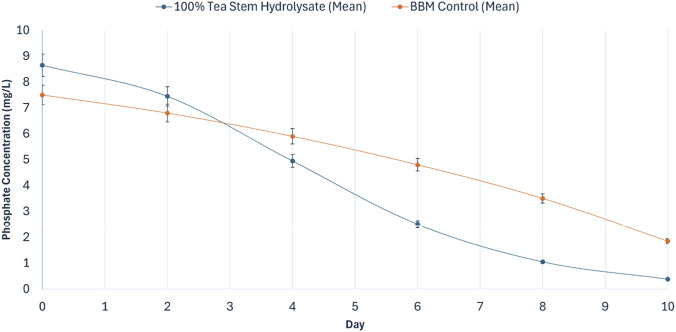
Temporal variation of phosphate-phosphorus (PO_4_^3-^–P, mg L^-1^) during main cultivation of *Chlorella vulgaris* in optimized tea stem hydrolysate (100% v/v, PPFD = 240 µmol photons m^-2^ s^-1^) compared with BBM control (PPFD = 140 µmol photons m^-2^ s^-1^). Values are mean ± SD (*n* = 3).

**FIGURE 9:**
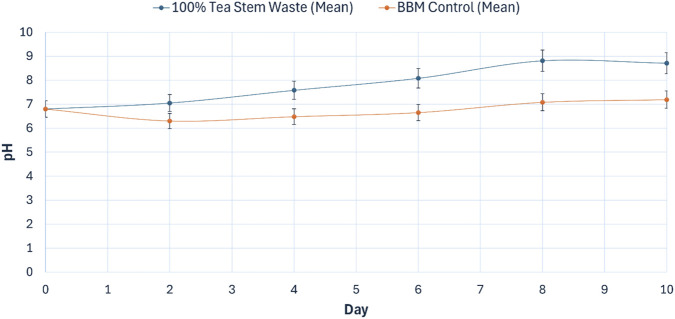
pH evolution during main cultivation of *Chlorella vulgaris* in optimized tea stem hydrolysate (100% v/v, PPFD = 240 µmol photons m^-2^ s^-1^) compared with BBM control (PPFD = 140 µmol photons m^-2^ s^-1^). Values are mean ± SD (*n* = 3).

Nutrient assimilation dynamics during cultivation were evaluated by monitoring nitrate (NO_3_^−^–N), phosphate (PO_4_^3-^–P), and pH over the batch period. As established during optimization, the tea stem hydrolysate was maintained at 240 µmol photons m^-2^ s^-1^, while the BBM control was maintained at a baseline of 140 µmol photons m^-2^ s^-1^. Temporal profiles are presented in Figures 7–9, and removal efficiencies were calculated relative to the initial concentrations measured at Day 0 of cultivation. It should be noted that the initial dissolved nutrient concentrations at Day 0 (NO_3_^−^–N: 100.18 ± 4.52 mg L^-1^; PO_4_^3-^–P: 8.65 ± 0.55 mg L^-1^) were slightly lower than the raw hydrolysate characterized in Table 1. This minor reduction is a standard artifact of the autoclave sterilization process, which frequently induces partial volatilization of nitrogenous compounds and the thermal precipitation of insoluble metal-phosphate complexes prior to inoculation.

#### 3.4.1 Nitrate (NO_3_^−^–N) uptake

The temporal variation in nitrate concentration is shown in Figure 7. The tea stem hydrolysate exhibited a higher initial NO_3_^−^–N concentration (100.18 ± 4.52 mg L^-1^) than the BBM control (41.50 ± 2.10 mg L^-1^). A rapid decline in NO_3_^−^–N was observed in the hydrolysate culture during the exponential growth window (Day 2–Day 8), decreasing from 85.44 mg L^-1^–29.25 mg L^-1^, consistent with the rapid biomass proliferation observed under optimized conditions. By Day 10, residual NO_3_^−^–N in the hydrolysate culture decreased to 16.80 ± 3.85 mg L^-1^, corresponding to a nitrate removal efficiency of 83.23%. In contrast, the BBM control exhibited slower uptake kinetics, reaching 18.20 ± 1.50 mg L^-1^ by Day 10 (56.14% removal). Endpoint comparisons confirmed significantly higher nitrate removal in the hydrolysate culture relative to BBM (*p* < 0.05).

#### 3.4.2 Phosphate (PO_4_^3-^–P) uptake

Phosphate uptake kinetics (Figure 8) followed a similar pattern. The initial PO_4_^3-^–P concentration in the tea stem hydrolysate was 8.65 ± 0.55 mg L^-1^, which declined to 2.50 mg L^-1^ by Day 6. By Day 10, phosphate was reduced to 0.38 ± 0.08 mg L^-1^, yielding a removal efficiency of 95.60%. The BBM control exhibited a lower removal performance, with a final phosphate concentration of 1.85 ± 0.15 mg L^-1^ (75.33% removal). Endpoint comparisons confirmed significantly higher phosphate removal in the hydrolysate culture (*p* < 0.05). The significant depletion of extracellular phosphate to 0.38 mg L^-1^, coupled with the reduction of nitrate to 16.80 mg L^-1^ by Day 10, indicates the onset of pronounced nutrient limitation. While absolute nutrient starvation (0 mg L^-1^) was not reached, this severe limitation represents a critical threshold known to induce metabolic shifts during the stationary phase, redirecting carbon flux toward enhanced lipid accumulation in oleaginous microalgae.

#### 3.4.3 pH evolution

The pH trajectories are summarized in Figure 9. Both media were initiated at pH 6.80. During cultivation, the tea stem hydrolysate culture exhibited pronounced alkalinization, increasing to 8.81 ± 0.15 b y Day 8 and stabilizing at 8.71 ± 0.15 b y Day 10. In comparison, the BBM control showed a modest increase, reaching 7.19 ± 0.12 b y Day 10. The higher pH in the hydrolysate culture is driven by two primary mechanisms. First, the uptake of dissolved inorganic carbon during rapid photosynthesis shifts the carbonate equilibria, increasing medium alkalinity. Second, the active co-assimilation of nitrate (NO_3_^−^) inherently involves the extrusion of hydroxyl ions (OH^−^) or the co-transport of protons (H^+^) by the microalgal cells to maintain intracellular charge balance, which significantly contributes to the observed alkalinization of the medium. Endpoint pH differed significantly between treatments (*p* < 0.05).

### 3.5 Lipid production and accumulation

To evaluate biodiesel-relevant performance beyond gross biomass formation, intracellular lipid accumulation was quantified during the transition from the late exponential to the stationary phase (Days 10–12). Volumetric lipid concentration (g L^-1^) was determined for the optimized tea stem hydrolysate culture and the BBM control, and the comparative results are summarized in Table 2.

**TABLE 2:** Temporal variation of volumetric lipid concentration in *Chlorella vulgaris* cultivated in 100% tea stem hydrolysate (PPFD = 240 µmol photons m^-2^ s^-1^) versus BBM control (PPFD = 140 µmol photons m^-2^ s^-1^) during stationary phase (Days 10–12) (mean ± SD, *n* = 3).

Cultivation day	Medium	Volumetric lipid concentration (g L^-1^)	Fold increase vs. control
Day 10	BBM (control)	0.021 ± 0.001	–
	Tea stem hydrolysate	0.087 ± 0.003	4.1×
Day 11	BBM (control)	0.021 ± 0.001	–
	Tea stem hydrolysate	0.089 ± 0.004	4.2×
Day 12	BBM (control)	0.021 ± 0.001	–
	Tea stem hydrolysate	0.094 ± 0.005	4.5×

The hydrolysate culture exhibited substantially higher lipid concentrations than the BBM control throughout the assessed stationary-phase interval. On Day 10, volumetric lipid concentration in the hydrolysate culture reached 0.087 ± 0.003 g L^-1^, compared with 0.021 ± 0.001 g L^-1^ in the BBM control (*p* < 0.001). Lipid concentration in the hydrolysate culture increased further, peaking on Day 12 at 0.094 ± 0.005 g L^-1^, whereas the BBM control remained low and relatively unchanged (∼0.021 g L^-1^). Consequently, at peak accumulation the hydrolysate system achieved approximately 4.5-fold higher volumetric lipid concentration than the synthetic control (*p* < 0.001).

The timing of enhanced lipid accumulation coincided with the nutrient-limited conditions described in Section 3.4 (residual NO_3_^−^–N: 16.80 ± 3.85 mg L^-1^; PO_4_^3-^–P: 0.38 ± 0.08 mg L^-1^ by Day 10). These conditions are consistent with widely reported nutrient-stress responses in oleaginous microalgae, where cellular carbon partitioning can shift away from protein synthesis and toward the biosynthesis of energy-dense neutral lipids (triacylglycerols) during stationary phase. Overall, the results demonstrate that tea stem hydrolysate cultivation significantly improves volumetric lipid output relative to BBM, supporting its potential as a low-cost cultivation medium for biodiesel-oriented bioprocessing.

### 3.6 Fatty acid profiling and biodiesel property prediction

The ultimate thermophysical quality, combustion efficiency, and storage stability of microalgal biodiesel are intrinsically governed by the fatty acid methyl ester (FAME) profile of the parent lipid. The FAME composition of *Chlorella vulgaris* cultivated in 100% tea stem hydrolysate was determined by GC–FID analysis (Table 3). Based on the measured FAME distribution, key biodiesel-relevant fuel properties were predicted using established empirical correlations (Table 4).

**TABLE 3:** Fatty acid profile of *Chlorella vulgaris* cultivated in 100% tea stem hydrolysate (normalized % of total FAMEs).

Fatty acid	Carbon structure	Relative abundance (%)
Caprylic acid	C8:0	2.05
Capric acid	C10:0	2.67
Lauric acid	C12:0	11.70
Myristic acid	C14:0	11.88
Myristoleic acid	C14:1	1.12
Palmitic acid	C16:0	34.62
Stearic acid	C18:0	21.96
Oleic acid	C18:1	6.05
Linoleic acid	C18:2	4.31
α-Linolenic acid	C18:3	2.90
**Total saturated**	–	**85.57**
**Total unsaturated**	–	**14.38**

Bold values indicate the major fatty acid components.

**TABLE 4:** Estimated fuel properties of *C. vulgaris* biodiesel derived from tea stem hydrolysate compared with ASTM D6751 and EN 14214 limits.

Property	Unit	This study (estimated)	ASTM D6751	EN 14214
Iodine value (IV)	g I_2_/100 g	22.44	–	≤120
Saponification value (SV)	mg KOH/g	226.52	–	–
Cetane number (CN)	–	65.35	≥47	≥51
Higher heating value (HHV)	MJ/kg	39.81	–	–
Oxidative stability (OS)	h	12.03	≥3	≥8

#### 3.6.1 FAME composition

The chromatographic analysis revealed a distinct lipid profile exhibiting a pronounced dominance of saturated fatty acids (SFAs), accounting for 85.57% of total FAMEs, while unsaturated fatty acids represented 14.38%. Palmitic acid (C16:0) was the most abundant component (34.62%), followed by stearic acid (C18:0, 21.96%). Medium-chain SFAs were also substantial, including lauric acid (C12:0, 11.70%) and myristic acid (C14:0, 11.88%), with smaller contributions from caprylic (C8:0, 2.05%) and capric acids (C10:0, 2.67%). Among the unsaturated fractions, oleic acid (C18:1) was 6.05%, linoleic acid (C18:2) 4.31%, and α-linolenic acid (C18:3) 2.90%.

This fatty acid distribution is consistent with reported physiological shifts under nutrient-limited and complex organic cultivation conditions, where microalgae may preferentially accumulate stable, energy-dense neutral lipids rather than polyunsaturated membrane-associated lipids. Overall, the high SFA fraction and comparatively low polyunsaturated fatty acid (PUFA) content (C18:2 + C18:3 = 7.21%) indicate a lipid profile favorable for oxidative stability and ignition quality, while potentially requiring attention to cold-flow behavior due to elevated long-chain saturation.

#### 3.6.2 Estimated biodiesel fuel properties and comparison to standards

Fuel properties were predicted from the FAME profile using the correlations described in Section 2.8. The calculated iodine value (IV), a key metric of unsaturation and oxidative stability, was 22.44 g I_2_/100 g, which is well below the EN 14214 maximum limit (≤120), reflecting the low degree of unsaturation. The estimated cetane number (CN) was 65.35, exceeding the minimum requirements for both ASTM D6751 (≥47) and EN 14214 (≥51), consistent with the high proportion of saturated and monounsaturated chains.

The predicted oxidative stability (OS) was 12.03 h, meeting both ASTM D6751 (≥3 h) and EN 14214 (≥8 h) requirements. In addition, the saponification value (SV) and higher heating value (HHV) were 226.52 mg KOH/g and 39.81 MJ/kg, respectively, indicating favorable energy density.

Cold-flow properties (e.g., cloud point and cold filter plugging point) and kinematic viscosity depend strongly on chain length and saturation. The abundance of long-chain saturated fatty acids (notably C16:0 and C18:0) can elevate the melting point and may adversely affect low-temperature operability. Because these parameters are best confirmed experimentally, they were not asserted beyond the scope of the applied prediction framework. Consequently, while this FAME profile suggests a highly stable fuel with strong ignition quality, it may be particularly well-suited as a premium blending stock to enhance the cetane rating and oxidative stability of conventional diesel or more unsaturated biodiesel blends.

## 4 Discussion

### 4.1 Biomass enhancement and mixotrophic potential in hydrolysate media

This study demonstrates that acid-hydrolyzed tea stem waste can serve as a functional cultivation medium for *Chlorella vulgaris*, enabling high-density biomass formation alongside substantial nutrient uptake and enhanced lipid accumulation under batch operation. The hydrolysate provided sufficient macronutrients to sustain rapid proliferation, while its dark and turbid character imposed a light-shading constraint that was mitigated through elevated irradiance. As cultivation progressed, depletion of bioavailable nitrogen and phosphate coincided with the transition into stationary phase and a measurable increase in volumetric lipid concentration, culminating in a FAME profile associated with high ignition quality and oxidative stability. Collectively, these findings support tea stem waste valorization within a circular biorefinery framework in which an underutilized lignocellulosic by-product is converted into algal biomass and biodiesel-relevant lipids while simultaneously reducing nutrient loads.

Compared with BBM, the tea stem hydrolysate supported substantially higher biomass formation under optimized conditions. Although direct literature on tea stem hydrolysate specifically outperforming BBM for *C. vulgaris* remains limited, the observed response aligns strongly with broader evidence that organic-rich lignocellulosic and agro-waste hydrolysates frequently promote superior *Chlorella* growth relative to synthetic media, primarily by enabling mixotrophic metabolism through the simultaneous availability of inorganic nutrients and soluble organic carbon (Lau et al., 2014; Arora and Philippidis, 2021). For instance, Lau et al. (2014) demonstrated that food-waste-derived substrates can sustain strong growth kinetics in *C. vulgaris* under carbon-rich conditions, while Arora and Philippidis (Arora and Philippidis, 2021) reported that sweet sorghum bagasse hydrolysate can support enhanced *C. vulgaris* biomass formation when hydrolysate loading is appropriately optimized. Similarly, recent circular biorefinery approaches have demonstrated that acid-hydrolyzed fishery waste significantly enhances both biomass and high-value pigment yields in *Spirulina* sp. Compared to synthetic BG-11 media, underscoring the broad versatility of waste-derived hydrolysates for mixotrophic cultivation (Dodangodage et al., 2026d) Collectively, these studies support the interpretation that hydrolysate-based media can outperform BBM by broadening the metabolic substrate spectrum beyond inorganic nutrients alone (Lau et al., 2014; Arora and Philippidis, 2021).

From a mechanistic perspective, the biomass enhancement observed in this study is consistent with the action of thermochemical depolymerization, which can mobilize structurally bound nitrogen and phosphorus and release soluble organics from lignocellulosic matrices into the aqueous phase (Wang et al., 2014; Zhou et al., 2025). However, lignocellulosic hydrolysates may also contain inhibitory by-products generated during acid hydrolysis (e.g., furanics and organic acids) that can suppress algal growth if concentrations exceed strain tolerance (Arora and Philippidis, 2021; Wang et al., 2014). Therefore, the absence of growth suppression at higher hydrolysate loadings in the present screening suggests that the applied hydrolysis and conditioning conditions produced a biologically compatible medium for *C. vulgaris* across the tested concentration range, indicating a favorable balance between nutrient/organic release and inhibitor formation.

A further plausible contributor to the superior performance is the micronutrient background inherently present in tea biomass. Tea plants are documented accumulators of several mineral elements, and acid hydrolysis may mobilize trace metals (e.g., Fe, Mg, Mn, Zn) that act as essential cofactors in photosynthetic electron transport and lipid metabolism, potentially reducing reliance on expensive, chelated trace-metal supplementation typical of synthetic media. While this mechanism remains a hypothesis in the absence of elemental profiling, it is consistent with the well-established micronutrient dependence of algal growth and warrants confirmation through ICP-based elemental analysis alongside DOC/TOC characterization in future work.

From an applied standpoint, the ability to cultivate *C. vulgaris* in 100% hydrolysate without further freshwater dilution beyond the initial hydrolysis preparation is industrially meaningful. Many waste-derived media exhibit substrate inhibition or require dilution due to inhibitory compounds or optical limitations at high loadings (Arora and Philippidis, 2021). In contrast, the present results indicate that tea stem hydrolysate can be used at full strength under optimized irradiance, thereby maximizing waste valorization while minimizing freshwater consumption, factors that directly enhance the scalability and sustainability of the proposed bioprocess. However, an important limitation must be acknowledged: the “100%” hydrolysate in this study was generated by diluting the initial filtrate to a 1.5 L working volume. While this represented the highest concentration evaluated here, the absolute limit of concentration was not established. Future research should explore the feasibility of working with even more concentrated hydrolysates (e.g., by diluting the initial filtrate to 1.0 L or 0.5 L) to determine the ultimate threshold of substrate toxicity and further minimize freshwater requirements.

### 4.2 Optical attenuation, irradiance optimization, and scale-up implications

Despite its nutrient richness, the tea stem hydrolysate exhibited strong optical attenuation, which can substantially reduce the effective photosynthetic photon flux density (PPFD) available within the culture volume. This phenomenon is widely reported for lignocellulosic hydrolysates and waste-derived media, where dissolved organics, pigments, and suspended colloids increase turbidity and absorb/scatter incident light, thereby limiting photon penetration even when macronutrients are abundant (Arora and Philippidis, 2021; Indrayani et al., 2023; Lyu et al., 2025). Consistent with this established behavior, the pronounced dependence of biomass accumulation on incident PPFD observed in this study indicates that light availability was the dominant rate-limiting factor during hydrolysate-based cultivation. Increasing PPFD to 240 µmol photons m^-2^ s^-1^ markedly improved both spectrophotometric growth profiles and gravimetric DCW, supporting the selection of elevated irradiance for the main cultivation phase. Similar PPFD-dependent growth responses have been reported for *Chlorella* spp., where higher irradiance promotes higher OD and DCW relative to lower illumination regimes under otherwise comparable operational conditions (Indrayani et al., 2023; Schnurr et al., 2016; Dhanasekar and Sathyanathan, 2023).

From a mechanistic perspective, elevated irradiance can compensate for attenuation in dark, colored matrices by increasing photon availability in deeper regions of the reactor and improving the probability that cells experience periods above the local light compensation point. However, excessive surface irradiance can also increase the risk of photoinhibition and photo-oxidative stress, particularly under suboptimal mixing where cells remain exposed to high light for prolonged intervals (García-Robledo et al., 2012; Panahi et al., 2019). In the present system, the absence of growth suppression at the highest evaluated PPFD is consistent with the concept that the hydrolysate matrix itself acts as a partial “optical buffer,” reducing the effective internal irradiance through absorption and scattering and thereby lowering the photoinhibition risk at the cell level.

However, mitigating this optical limitation introduces a complex trade-off that must be critically evaluated through a Life Cycle Assessment (LCA) perspective. On one hand, utilizing highly concentrated waste streams is beneficial for the water consumption aspect of the LCA, as it drastically reduces the freshwater footprint of the bioprocess. On the other hand, a more concentrated hydrolysate fundamentally increases medium turbidity and color intensity. At larger scales, this inherent turbidity is further compounded by mutual cell shading and complex light–dark cycling, which inherently cap volumetric productivity (Akca et al., 2024; Kumar et al., 2015). To maintain baseline photosynthetic performance in such dense media, significantly higher levels of incident irradiance are required. As highlighted in recent scale-up studies, photon delivery often becomes the primary energetic bottleneck when utilizing dark, hydrolysate-rich cultivation matrices (Hoeniges et al., 2022; Ren et al., 2025). This forced increase in irradiance has a direct, negative impact on the energy consumption aspect of the LCA. Consequently, any industrial scale-up of this process cannot simply maximize waste concentration; it must critically evaluate the optical parameters to find the optimal balance between minimizing the freshwater footprint and minimizing the electrical energy required for photon delivery. Furthermore, while 240 µmol photons m^-2^ s^-1^ yielded the highest productivity in this study, the absolute upper threshold of irradiance was not established; future research should investigate whether even higher light intensities could further enhance biomass yields without inducing photoinhibition.

### 4.3 Nutrient uptake, secondary metabolites, and lipid accumulation

Temporal nutrient profiles indicated rapid assimilation of nitrate and significant depletion of phosphate by Day 10 under tea stem hydrolysate cultivation, coinciding with peak biomass and the onset of stationary phase. Such nutrient limitation is widely recognized as a principal trigger for metabolic reprogramming in oleaginous microalgae, where cellular carbon partitioning shifts from growth-associated protein synthesis toward the accumulation of energy-dense neutral lipids, primarily triacylglycerols (TAGs). Under nitrogen limitation, *Chlorella* typically downregulates amino acid and protein biosynthesis, while redirecting carbon flux through acetyl-CoA toward fatty acid and TAG formation; phosphate limitation can further intensify this response by constraining ATP-dependent anabolic pathways and altering lipid class distribution. Therefore, the observed increase in lipid concentration during Days 10–12, and the ∼4.5-fold higher volumetric lipid yield relative to the BBM control, is consistent with a classic nutrient-stress-driven lipid accumulation trajectory in *C. vulgaris*.

Beyond macronutrient limitation, the tea stem hydrolysate matrix may also impose additional biochemical cues that modulate lipid metabolism. Tea stems from *Camellia sinensis* are known to contain secondary metabolites such as polyphenols and tannins, which can leach into process streams and exhibit concentration-dependent biological effects. Recent studies have shown that tea-related phenolic compounds can influence microalgal physiology in a biphasic manner, where low-to-moderate exposure stimulates cellular metabolism and lipid accumulation without necessarily suppressing photosynthetic performance, whereas higher concentrations can inhibit growth through oxidative stress and cellular disruption (Zheng et al., 2023; Zou et al., 2023; Huang and Zhang, 2024). This dose-dependent behavior is consistent with hormesis, a well-established stress-physiology concept in microalgae wherein mild abiotic stress can activate adaptive signaling (including ROS-mediated regulation) and increase storage lipid accumulation (Su et al., 2020; Zou L. et al., 2023; Shi et al., 2017; Shi et al., 2020). In this context, the simultaneous occurrence of nutrient limitation and the complex organic/secondary-metabolite composition of tea stem hydrolysate could plausibly act in combination to precondition cells toward stationary-phase TAG biosynthesis.

However, because phenolic content, speciation, and degradation kinetics were not directly measured in this study, any specific attribution of the lipid increase to tea-derived polyphenols or tannins should be treated as a testable hypothesis rather than a confirmed mechanism. Future work should quantify total phenolics and identify dominant compounds via HPLC-based profiling, coupled with controlled dose–response assays to define inhibitory thresholds and determine whether phenolic exposure synergistically amplifies nutrient-stress-induced lipogenesis in *C. vulgaris*. The present results indicate that tea stem hydrolysate can deliver a favorable “growth-to-lipid” transition within a single waste-derived medium, driven primarily by nutrient limitation and potentially modulated by the matrix’s secondary-metabolite composition, thereby reducing reliance on a separate stress-induction step.

### 4.4 FAME profiling and estimated biodiesel fuel properties

The FAME profile obtained under tea stem hydrolysate cultivation was dominated by saturated fatty acids (SFAs), particularly C16:0 and C18:0, with comparatively low PUFA content. Similar SFA-dominant patterns, often with palmitic acid in the ∼20–40% range and stearic acid in the ∼10–27% range, have been reported when microalgae are cultivated under carbon-rich heterotrophic or mixotrophic regimes, including cultivation on complex waste or hydrolysate-derived substrates (Rohit and Venkata Mohan, 2018; Irmak and Arzu, 2020; Fruit Wastes Hydrolysates as Feedstock, 2017). Consistent with established structure–property relationships for SFA-enriched oils, the predicted fuel properties fall within the acceptable thresholds specified by ASTM D6751 and EN 14214 standards, confirming the suitability of the produced biodiesel. Furthermore, the use of organic carbon-rich residues and alternative hydrolysates has been widely shown to improve neutral lipid productivity in *C. vulgaris* under mixotrophic conditions, yielding FAME distributions highly compatible with commercial biodiesel targets (Abomohra et al., 2021; Qi et al., 2020).

Nevertheless, a key trade-off of SFA-rich profiles is the potential deterioration of cold-flow properties. Increased long-chain saturation promotes crystallization at higher temperatures, elevating cloud point and worsening cold filter plugging point (CFPP), which can restrict direct use in colder climates or seasonal conditions (Dunn, 2010; Bouaid et al., 2024). Because cold-flow behavior is strongly influenced by the detailed ester distribution and crystallization kinetics, it should be validated experimentally using standardized methods (e.g., ASTM D2500 for cloud point and ASTM D6371 for CFPP) rather than asserted solely from profile-based prediction (Dunn, 2010; Bouaid et al., 2024). In this context, the present biodiesel is likely best positioned as a premium blending stock: SFA-rich esters can enhance CN and oxidative stability when blended into more unsaturated biodiesel streams or petroleum diesel, improving storage life and ignition quality while partially moderating cold-flow penalties at practical blend ratios (Dunn, 2010; Hazrat et al., 2020; Longanesi et al., 2022). Overall, the FAME composition observed under tea stem hydrolysate cultivation supports strong fuel stability and ignition quality, with cold-flow validation and blending optimization representing clear next steps for practical deployment.

### 4.5 Pretreatment intensity, biorefinery integration, and future priorities

An objective assessment of industrial feasibility must explicitly account for pretreatment intensity. Acid hydrolysis using sulfuric acid under elevated temperature is a highly effective approach for disrupting lignocellulosic structure and mobilizing soluble nutrients and carbon; however, it introduces non-trivial thermal and chemical demands that can impose a measurable energy penalty if the process is not integrated and optimized (Karthikeyan, 2023; Oliveira et al., 2025). Recent sustainability analyses of thermochemical pretreatment routes emphasize that pretreatment can become a dominant energy contributor in biomass-to-fuels value chains, potentially reducing net energy balance and weakening metrics such as energy return on investment (EROI) when assessed against the recoverable energy content of biodiesel and co-products (Oliveira et al., 2025; de Farias Silva et al., 2020). Although a full energy balance was beyond the scope of this study, the high biomass and lipid productivity observed here suggests potential to offset pretreatment energy inputs when integrated within a biorefinery framework. Accordingly, future work should quantify the full energy inventory of hydrolysis, neutralization, and sterilization steps and evaluate net energy performance relative to the higher heating value (HHV) of the biodiesel fraction and any recoverable co-products, ideally using integrated process simulation coupled with life cycle assessment (Oliveira et al., 2025).

In practical industrial deployments, the pretreatment energy penalty may be substantially mitigated through process integration. Tea processing facilities typically generate low-to medium-grade waste heat, and coupling hydrolysis operations with waste heat recovery or heat-exchanger networks could significantly reduce external thermal energy demand. Such integration strategies are increasingly recognized as essential for achieving favorable sustainability performance in hydrolysis-based biorefineries (Oliveira et al., 2025). In addition, process intensification options (e.g., optimizing acid concentration, residence time, and solids loading) can reduce chemical and heating requirements while minimizing inhibitor formation, thereby improving the likelihood of a positive net energy balance (Karthikeyan, 2023; de Farias Silva et al., 2020).

Beyond energy balance, the economic viability of microalgal biodiesel systems typically depends on implementing a true biorefinery approach, because biodiesel is a low-margin, high-volume product. Consequently, valorization of lipid-extracted algae (LEA), the residual biomass remaining after lipid recovery, is critical for improving carbon efficiency and generating additional revenue streams (Laurens et al., 2017; Tsarpali et al., 2021). The LEA retains substantial protein- and carbohydrate-rich fractions that can be upgraded via multiple routes, including anaerobic digestion for biogas (methane), fermentation feedstock applications, or thermochemical conversion to produce biochar or hydrochar (Tsarpali et al., 2021; Yadav et al., 2022). Beyond traditional bioenergy co-products, algal biomass and its derivative fractions are increasingly being explored for the development of advanced sustainable biomaterials, such as algae-based protective coatings for green infrastructure, further expanding the techno-economic portfolio of the biorefinery (Dodangodage et al., 2025b). These co-product pathways substantially improve overall process circularity and strengthen techno-economic feasibility (Laurens et al., 2017; Kumar et al., 2022). From a regional sustainability perspective, thermochemical conversion of LEA to biochar may be particularly attractive for tea cultivation systems, as biochar amendments can enhance soil organic carbon retention, improve nutrient holding capacity, and contribute to long-term carbon sequestration in plantation soils (Yadav et al., 2022). Therefore, coupling tea stem hydrolysate cultivation with LEA valorization is a central design requirement for establishing a circular, industrially realistic algal biorefinery that offsets pretreatment energy penalties (Oliveira et al., 2025; Laurens et al., 2017; Tsarpali et al., 2021).

Several limitations and future priorities emerge from this work. First, cultivation was performed in batch mode; continuous or fed-batch strategies could sustain higher biomass productivity while managing nutrient depletion trajectories and potentially enabling controlled two-stage operation (growth followed by lipid induction). Second, hydrolysate composition may vary spatially and seasonally, requiring robust quality control and adaptive dilution/conditioning strategies. Third, outdoor performance under fluctuating sunlight and temperature should be tested to assess stability beyond controlled LED conditions. Finally, while sterilization was used here to ensure experimental consistency, industrial-scale cultivation is typically non-sterile; tea-derived antimicrobial secondary metabolites may offer a potential advantage for contamination suppression, but this should be validated explicitly through mixed-culture challenge tests rather than assumed.

Furthermore, while the advantages of waste-derived cultivation are substantial, the transition from laboratory to industrial deployment faces significant practical and regulatory hurdles. Industrial waste streams are inherently subject to seasonal and compositional variability, meaning the nutrient load, pH, and inhibitor concentrations of the hydrolysate will fluctuate. This lack of standardization complicates continuous bioprocess control. Additionally, utilizing agro-industrial waste introduces regulatory challenges regarding heavy metal accumulation, pesticide residues, and environmental discharge standards. If the resulting biodiesel or co-products (such as LEA biochar) contain concentrated contaminants derived from the raw waste, they may fail to meet stringent commercial quality and environmental compliance thresholds.

From a broader sustainability perspective, the observed nutrient uptake suggests that waste-derived cultivation can partially offset the need for refined nutrient inputs typically associated with synthetic media. Replacing a portion of fertilizer-derived nitrogen and phosphorus inputs may reduce upstream embodied energy and emissions, strengthening the life-cycle performance of microalgal biodiesel. Future studies should therefore integrate process data into life cycle assessment and techno-economic models to quantify carbon intensity, cost drivers, and practical deployment scenarios. This is especially critical in tea-producing regions where waste disposal and diesel import dependence are persistent challenges. This requirement for rigorous life cycle validation aligns with a growing cross-disciplinary consensus, spanning from biochemical bioprocesses to sustainable construction materials, that the true environmental benefits of circular waste valorization must be explicitly quantified to justify industrial deployment (Fernando et al., 2026).

## 5 Conclusion

This study demonstrates that acid-hydrolyzed tea stem waste can be effectively valorized as a low-cost, high-efficiency cultivation medium for *C. vulgaris* within an integrated biorefinery framework. Thermochemical hydrolysis produced a nutrient-rich, bioavailable matrix that, when coupled with optimized irradiance to overcome light attenuation, supported robust mixotrophic growth and rapid nutrient remediation. The undiluted hydrolysate significantly outperformed synthetic media (e.g., higher biomass and lipid yields), functioning as an efficient integrated cultivation matrix. Importantly, nutrient limitation during the late cultivation phase acted as a natural abiotic stressor, inducing lipid accumulation without the need for a separate stress-induction step. This resulted in a saturated fatty acid profile associated with high oxidative stability and ignition quality, yielding a biodiesel suitable for premium blending applications and meeting key fuel property requirements.

Overall, this work advances a circular bioeconomy pathway by integrating lignocellulosic waste valorization with renewable bioenergy production. By enabling high-density biomass cultivation while reducing environmental nutrient loads, this approach provides a scalable model for decentralized waste-to-energy systems. To support industrial translation, future research should focus on continuous cultivation strategies, scale-appropriate photobioreactor design for dark effluents under non-sterile outdoor conditions, and rigorous validation through techno-economic and life-cycle assessments. Additionally, further studies must establish the absolute upper thresholds for hydrolysate concentration (e.g., reducing the initial hydrolysis working volume) and incident irradiance to determine if even greater productivities can be achieved. This study highlights the potential of coupling agro-industrial waste streams with microalgal bioprocesses to develop resource-efficient and scalable biofuel production systems.

## Data Availability

The raw data supporting the conclusions of this article will be made available by the authors, without undue reservation.

## References

[B1] Abomohra A. E. F. Wang Q. Huang J. Saad-Allah K. M. (2021). A sustainable approach for bioconversion of food and lignocellulosic wastes into liquid biofuel using a new Metschnikowia pulcherrima isolate. Int. J. Energy Res. 45 (2), 3430–3441. 10.1002/er.6028

[B2] Adams C. Godfrey V. Wahlen B. Seefeldt L. Bugbee B. (2013). Understanding precision nitrogen stress to optimize the growth and lipid content tradeoff in oleaginous green microalgae. Bioresour. Technol. 131, 188–194. 10.1016/j.biortech.2012.12.143 23347926

[B3] Akca M. S. Kinaci O. K. Inanc B. (2024). Improving light availability and creating high-frequency light–dark cycles in raceway ponds through vortex-induced vibrations for microalgae cultivation: a fluid dynamic study. Bioprocess Biosyst. Eng. 47 (11), 1863–1874. 10.1007/s00449-024-03074-5 39133298 PMC11438835

[B4] Akondi R. N. Trexler R. V. Pfiffner S. M. Mouser P. J. Sharma S. (2017). Modified lipid extraction methods for deep subsurface shale. Front. Microbiol. 8 (JUL), 1408. 10.3389/fmicb.2017.01408 28790998 PMC5524817

[B5] Arora N. Philippidis G. P. (2021). Insights into the physiology of Chlorella vulgaris cultivated in sweet sorghum bagasse hydrolysate for sustainable algal biomass and lipid production. Sci. Rep. 11 (1), 6779. 10.1038/s41598-021-86372-2 33762646 PMC7991646

[B6] Association A. P. H. (1926). Standard methods for the examination of water and wastewater, 6. American public health association.

[B7] Bharti R. K. Kaushal C. Singh A. Dhar D. W. Babu R. Kaushik A. (2024). Evaluation of fuel properties for possible biodiesel output based on the fatty acid composition of oleaginous plants and microalgae. Sci. Total Environ. 918, 170448. 10.1016/j.scitotenv.2024.170448 38301774

[B8] Bischoff H. W. (1963). Bold HC some soil algae from enchanted rock and related algal species. Austin, TX: University of Texas, 1–95.

[B9] Bligh E. G. Justin Dyer W. (1959). A rapid method of total lipid extraction and purification. Can. Journal Biochemistry Physiology 37 (8), 911–917. 10.1139/o59-099 13671378

[B10] Bouaid A. Iliuta G. Marchetti J. M. (2024). Cold flow properties of biodiesel from waste cooking oil and a new improvement method. Heliyon 10 (17), e36756. 10.1016/j.heliyon.2024.e36756 39281653 PMC11401029

[B11] Brandenburg J. Poppele I. Blomqvist J. Puke M. Pickova J. Sandgren M. (2018). Bioethanol and lipid production from the enzymatic hydrolysate of wheat straw after furfural extraction. Appl. Microbiol. Biotechnol. 102 (14), 6269–6277. 10.1007/s00253-018-9081-7 29804136 PMC6013517

[B12] Breuer G. Evers W. A. C. de Vree J. H. Kleinegris D. M. M. Martens D. E. Wijffels R. H. (2013). Analysis of fatty acid content and composition in microalgae. J. Vis. Exp. JoVE 80, e50628. 10.3791/50628 24121679 PMC3938209

[B13] Çakmak T. G. Saricaoglu B. Ozkan G. Tomas M. Capanoglu E. (2024). Valorization of tea waste: composition, bioactivity, extraction methods, and utilization. Food Sci. and Nutr. 12 (5), 3112–3124. 10.1002/fsn3.4011 38726441 PMC11077253

[B14] Casanova L. M. Mendes L. B. B. Corrêa T. de S. da Silva R. B. Joao R. R. Macrae A. (2023). Development of microalgae biodiesel: current status and perspectives. Microorganisms 11 (Number 1), 34. 10.3390/microorganisms11010034 36677325 PMC9862501

[B15] Chen R. Wang Y. Z. Liao Q. Zhu X. Xu T. F. (2013). Hydrolysates of lignocellulosic materials for biohydrogen production. BMB Rep. 46 (5), 244–251. 10.5483/BMBRep.2013.46.5.038 23710634 PMC4133895

[B16] de Farias Silva C. E. Meneghello D. de Souza Abud A. K. Bertucco A. (2020). Pretreatment of microalgal biomass to improve the enzymatic hydrolysis of carbohydrates by ultrasonication: yield vs energy consumption. J. King Saud University-Science 32 (1), 606–613. 10.1016/j.jksus.2018.09.007

[B17] Dhanasekar S. Sathyanathan R. (2023). Bioenergy potential of Chlorella vulgaris under the influence of different light conditions in a bubble column photobioreactor.

[B18] Dodangodage C. A. Premarathne H. Nadeniya C. Nethsara Gamage G. Halwatura R. H. Kasturiarachchi J. C. (2025a). Mixotrophic cultivation of desmodesmus sp. in Matured compost leachate: growth kinetics, nutrient removal, and stress-induced lipid production. 10.20944/preprints202601.1662.v1

[B19] Dodangodage C. A. Premarathne H. Kasturiarachchi J. C. Perera T. A. Rajapakshe D. Halwatura R. U. (2025b). Algae-Based protective coatings for sustainable infrastructure: a novel framework linking material chemistry, techno-economics, and environmental functionality. Phycology 5 (4), 84. 10.3390/phycology5040084

[B20] Dodangodage C. A. Gamage G. N. Mallawa L. C. Kasturiarachchi J. C. Fernando K. V. Halwatura R. H. (2026a). Production of carbohydrate-rich chlorella sp. biomass using clarified aquaponics effluent for bioethanol feedstock applications. Biomass 6 (2), 26. 10.3390/biomass6020026

[B21] Dodangodage C. A. Kasturiarachchi J. C. Wijesekara I. A. Perera T. A. Rajapakshe D. Halwatura R. (2026b). Integrated microalgal–aquaponic systems for enhanced water treatment and food security: a critical review of recent advances in process integration and resource recovery. Phycology 6 (1), 14. 10.3390/phycology6010014

[B22] Dodangodage C. A. Gamage G. N. Wijesekara I. A. Kasturiarachchi J. C. Perera T. A. Rajapakshe D. (2026c). Valorization of canteen wastewater through optimized Spirulina platensis cultivation for enhanced carotenoid production and nutrient removal. Phycology 6 (1), 15. 10.3390/phycology6010015

[B23] Dodangodage C. A. Gamage G. N. Halwatura R. H. Kasturiarachchi J. C. Perera T. A. Rajapakshe S. D. (2026d). Circular valorization of acid-hydrolyzed fishery wastewater: integrated remediation and enhanced C-Phycocyanin production by spirulina sp. Front. Environ. Sci. 14, 1816065. 10.3389/fenvs.2026.1816065

[B24] Dunn R. (2010). Cold flow properties of biodiesel by automatic and manual analysis methods. J. ASTM Int. 7 (4), 1–15. 10.1520/jai102618

[B25] Energy Agency I. (2025). Global energy review 2025. Available online at: www.iea.org (Accessed March 24, 2024).

[B26] Ezhumalai G. Arun M. Manavalan A. Rajkumar R. Heese K. (2024). A holistic approach to circular bioeconomy through the sustainable utilization of microalgal biomass for biofuel and other value-added products. Microb. Ecol. 87 (Number 1), 61. 10.1007/s00248-024-02376-1 38662080 PMC11045622

[B27] Farouk S. M. Tayeb A. M. Abdel-Hamid S. M. S. Osman R. M. (2024). Recent advances in transesterification for sustainable biodiesel production, challenges, and prospects: a comprehensive review. Environ. Sci. Pollut. Res. 31, 12722–12747. Number 9. 10.1007/s11356-024-32027-4 38253825 PMC10881653

[B28] Fernando K. V. Dodangodage C. A. Seneviratne V. M. Jayasinghe S. M. Dharmaratne D. Gamage G. N. (2026). Circular valorization of post-industrial textile waste in thermal-insulating cementitious ceiling sheets. Textiles 6 (1), 27. 10.3390/textiles6010027

[B29] Fruit wastes hydrolysates as feedstock: pre-treatment strategies for cost-saving and sustainable microalgae cultivation. (2017). Int. J. Res. Environ. Sci., 3(2) 10.20431/2454-9444.0302004

[B30] Gao F. Yang H. L. Li C. Peng Y. Y. Lu M. M. Jin W. H. (2019). Effect of organic carbon to nitrogen ratio in wastewater on growth, nutrient uptake and lipid accumulation of a mixotrophic microalgae chlorella sp. Bioresour. Technol. 282, 118–124. 10.1016/J.BIORTECH.2019.03.011 30852331

[B31] García-Robledo E. Corzo A. Papaspyrou S. Morris E. P. (2012). Photosynthetic activity and community shifts of microphytobenthos covered by green macroalgae. Environ. Microbiol. Rep. 4 (3), 316–325. 10.1111/j.1758-2229.2012.00335.x 23760795

[B32] Harrison H. H. Watts J. L. (2022). “Lipid extraction and analysis,” in Methods and applications. Editors Haspel G. Hart A. C. elegans C. (Springer), 271–281. US. 10.1007/978-1-0716-2181-3_14 PMC11168205 35320570

[B33] Hazrat M. A. Rasul M. G. Mofijur M. Khan M. M. K. Djavanroodi F. Azad A. K. (2020). A mini review on the cold flow properties of biodiesel and its blends. In Front. Energy Res. (Vol. 8). 598651, 10.3389/fenrg.2020.598651

[B34] Hoeniges J. Welch W. Pruvost J. Pilon L. (2022). A novel external reflecting raceway pond design for improved biomass productivity. Algal Res. 65, 102742. 10.1016/J.ALGAL.2022.102742

[B35] Huang B. Zhang J. (2024). Secondary metabolism in tea plants: pathways and regulatory mechanisms. J.Tea Sci Res. 14 (6), 313–321. 10.5376/jtsr.2024.14.0029

[B36] Indrayani I. Ramadani N. Z. Mawaddah N. Kaseng E. S. Sukainah A. Putra R. P. (2023). “Influence of different culture media and light intensity on the growth and biomass productivity of a newly isolated chlorella sp,” in UNM-IND1 from waepella hot spring, South Sulawesi, Indonesia, 16. Available online at: http://www.bioflux.com.ro/aacl (Accessed June 24, 2024).

[B37] Irmak Ş. O. Arzu A.-B. (2020). Determination of the fatty-acid composition of four native microalgae species. GSC Adv. Res. Rev. 4 (1), 001–008. 10.30574/gscarr.2020.4.1.0053

[B38] Islam M. A. Magnusson M. Brown R. J. Ayoko G. A. Nabi M. N. Heimann K. (2013). Microalgal species selection for biodiesel production based on fuel properties derived from fatty acid profiles. Energies 6 (11), 5676–5702. 10.3390/en6115676

[B39] Istasse T. Richel A. (2020). Mechanistic aspects of saccharide dehydration to furan derivatives for reaction media design. RSC Adv. 10 (40), 23720–23742. 10.1039/d0ra03892j 35517323 PMC9055118

[B40] Kadier A. Ilyas R. A. Huzaifah M. R. M. Harihastuti N. Sapuan S. M. Harussani M. M. (2021). Polymers use of industrial wastes as sustainable nutrient sources for bacterial cellulose (BC) production: mechanism, advances, and future perspectives. 10.3390/polym PMC8512337 34641185

[B41] Kanwal F. Aslam A. Torriero A. A. J. (2025). Microalgae-based biodiesel: integrating AI, CRISPR and nanotechnology for sustainable biofuel development. Emerg. Top. Life Sci. 8 (3), 131–143. 10.1042/ETLS20240004 40982615 PMC12599237

[B42] Karthikeyan S. (2023). Large scale cultivation and pretreatment optimization of freshwater microalgae biomass for bioethanol production by yeast fermentation. Nat. Environ. Pollut. Technol. 22 (2), 1035–1040. 10.46488/NEPT.2023.v22i02.051

[B43] khalaji M. (2022). Evaluation of fatty acid profiles of Chlorella vulgaris microalgae grown in dairy wastewater for producing biofuel. J. Environ. Health Sci. Eng. 20 (2), 691–697. 10.1007/s40201-022-00808-z 36406613 PMC9672247

[B44] Kumar K. Mishra S. K. Shrivastav A. Park M. S. Yang J. W. (2015). Recent trends in the mass cultivation of algae in raceway ponds. Renew. Sustain. Energy Rev. 51, 875–885. 10.1016/j.rser.2015.06.033

[B45] Kumar L. Mohan L. Anand R. Joshi V. Chugh M. Bharadvaja N. (2022). A review on unit operations, challenges, opportunities, and strategies to improve algal based biodiesel and biorefinery. Front. Chem. Eng. 4, 998289. 10.3389/fceng.2022.998289

[B46] Lau K. Y. Pleissner D. Lin C. S. K. (2014). Recycling of food waste as nutrients in Chlorella vulgaris cultivation. Bioresour. Technol. 170, 144–151. 10.1016/j.biortech.2014.07.096 25128844

[B47] Laurens L. M. L. Markham J. Templeton D. W. Christensen E. D. Van Wychen S. Vadelius E. W. (2017). Development of algae biorefinery concepts for biofuels and bioproducts; a perspective on process-compatible products and their impact on cost-reduction. Energy Environ. Sci. 10 (8), 1716–1738. 10.1039/c7ee01306j

[B48] Lee Y.-K. Chen W. Shen H. Han D. Li Y. Jones H. D. T. (2013). Basic culturing and analytical measurement techniques. In Handbook of microalgal culture (pp. 37–68). 10.1002/9781118567166.ch3

[B49] Liu T. Li Y. Liu F. Wang C. (2016). The enhanced lipid accumulation in oleaginous microalga by the potential continuous nitrogen-limitation (CNL) strategy. Bioresour. Technol. 203, 150–159. 10.1016/j.biortech.2015.12.021 26724547

[B50] Liu Y. Liu X. Zhang L. Xiao P. Qiu F. Cheng Z. (2025). A review of strategies enhancing lipid production from chlorella: progress and comparative analysis. Sustain. Energy Technol. Assessments 74, 104190. 10.1016/j.seta.2025.104190

[B51] Longanesi L. Pereira A. P. Johnston N. Chuck C. J. (2022). Oxidative stability of biodiesel: recent insights. Biofuels, Bioprod. Biorefining 16 (1), 265–289. 10.1002/bbb.2306

[B52] Lyu Q. Dar R. A. Baganz F. Smoliński A. Rasmey A. H. M. Liu R. (2025). Effects of lignocellulosic biomass-derived hydrolysate inhibitors on cell growth and lipid production during microbial fermentation of oleaginous microorganisms—A review. Fermentation 11 (Number 3), 121. (MDPI). 10.3390/fermentation11030121

[B53] Ma X. Liu J. Liu B. Chen T. Yang B. Chen F. (2016). Physiological and biochemical changes reveal stress-associated photosynthetic carbon partitioning into triacylglycerol in the oleaginous marine alga Nannochloropsis oculata. Algal Res. 16, 28–35. 10.1016/J.ALGAL.2016.03.005

[B54] Mallick N. Mandal S. Singh A. K. Bishai M. Dash A. (2012). Green microalga *Chlorella vulgaris* as a potential feedstock for biodiesel. J. Chem. Technol. and Biotechnol. 87 (1), 137–145. 10.1002/jctb.2694

[B55] Mattos E. R. Singh M. Cabrera M. L. Das K. C. Program B. and C. C. (2012). Effects of inoculum physiological stage on the growth characteristics of Chlorella sorokiniana cultivated under different CO2 concentrations. Appl. Biochem. Biotechnol. 168 (3), 519–530. 10.1007/s12010-012-9793-6 22836749

[B56] Mondal M. Khan A. A. Halder G. (2021). Estimation of biodiesel properties based on fatty acid profiles of chlamydomonas sp. BTA 9032 and chlorella sp. BTA 9031 obtained under mixotrophic cultivation conditions. Biofuels. 12(10), 1175–1181. 10.1080/17597269.2019.1600453

[B57] Naseema Rasheed R. Pourbakhtiar A. Mehdizadeh Allaf M. Baharlooeian M. Rafiei N. Alishah Aratboni H. (2023). Microalgal co-cultivation-recent methods, trends in omic-studies, applications, and future challenges. Front. Bioeng. Biotechnol. 11, 1193424. 10.3389/fbioe.2023.1193424 37799812 PMC10548143

[B58] Nassar Y. Khaleel M. (2024). International journal of electrical engineering and sustainability (IJEES) sustainable development and the surge in electricity demand across emerging economies. Available online at: https://ijees.org/index.php/ijees/index (Accessed February 22, 2024).

[B59] Ni L. Wang J. Fang Y. Zhu C. Wizi J. Jiang Z. (2023). An innovative strategy to control microcystis growth using tea polyphenols sustained-release particles: preparation, characterization, and inhibition mechanism. Environ. Sci. Pollut. Res. 30 (15), 43113–43125. 10.1007/s11356-023-25255-7 36648729

[B60] Nicodemou A. Konstantinou D. Koutinas M. (2024). Enhanced biomass and lipid production from olive processing wastewater using Scenedesmus obliquus in a two-stage cultivation strategy under salt stress. Biochem. Eng. J. 205, 109290. 10.1016/j.bej.2024.109290

[B61] Oliveira A. P. de S. Assemany P. P. Jackeline de Siqueira C. de Jesus Júnior M. M. de Ávila Rodrigues F. de Oliveira L. F. C. (2025). Hydrothermal carbonization of microalgae biomass from wastewater treatment: effects of acid pretreatment. ACS Omega 10 (30), 33138–33148. 10.1021/acsomega.5c02582 40787395 PMC12332549

[B62] Palanisamy K. M. Bhuyar P. Rahim M. A. Vadiveloo A. Al-Dhabi N. A. Govindan N. (2023). Lipid enhancement in oleaginous nannochloropsis sp. under nitrate limitation for future bioenergy production. Int. J. Energy Res. 2023 (1), 5412660–5412668. 10.1155/2023/5412660

[B63] Panahi Y. Khosroshahi A. Y. Sahebkar A. Heidari H. R. (2019). Impact of cultivation condition and media content on Chlorella vulgaris composition. Adv. Pharm. Bull. 9 (2), 182–194. 10.15171/apb.2019.022 31380244 PMC6664117

[B64] Porninta K. Khemacheewakul J. Techapun C. Phimolsiripol Y. Jantanasakulwong K. Sommanee S. (2023). Pretreatment and enzymatic hydrolysis optimization of lignocellulosic biomass for ethanol, xylitol, and phenylacetylcarbinol co-production using Candida magnoliae. Front. Bioeng. Biotechnol. 11, 1332185. 10.3389/fbioe.2023.1332185 38304106 PMC10830760

[B65] Qi F. Shen P. Hu R. Xue T. Jiang X. Qin L. (2020). Carotenoids and lipid production from Rhodosporidium toruloides cultured in tea waste hydrolysate. Biotechnol. Biofuels 13 (1), 74. 10.1186/s13068-020-01712-0 32322304 PMC7161300

[B66] Ren S. Shao C. Zhu F. Schagerl M. Hu X. Sobhi M. (2025). Optimization and synergistic enhancement of microalgae productivity in laboratory raceway ponds *via* co-regulation of automated light-supplemented mixers and electric field system. Biotechnol. Biofuels Bioprod. 18 (1), 63. 10.1186/s13068-025-02658-x 40517232 PMC12166589

[B67] Rohit M. V. Venkata Mohan S. (2018). Quantum yield and fatty acid profile variations with nutritional mode during microalgae cultivation. Front. Bioeng. Biotechnol. 6 (SEP), 111. 10.3389/fbioe.2018.00111 30320078 PMC6167444

[B68] Schnurr P. J. Molenda O. Edwards E. Espie G. S. Allen D. G. (2016). Improved biomass productivity in algal biofilms through synergistic interactions between photon flux density and carbon dioxide concentration. Bioresour. Technol. 219, 72–79. 10.1016/j.biortech.2016.06.129 27479797

[B69] Shi K. Gao Z. Shi T. Q. Song P. Ren L. J. Huang H. (2017). Reactive oxygen species-mediated cellular stress response and lipid accumulation in oleaginous microorganisms: the state of the art and future perspectives. Front. Microbiol. 8 (Number MAY), 793. 10.3389/fmicb.2017.00793 28507542 PMC5410592

[B70] Shi T.-Q. Wang L.-R. Zhang Z.-X. Sun X.-M. Huang H. (2020). Stresses as first-line tools for enhancing lipid and carotenoid production in microalgae. Front. Bioeng. Biotechnol. 8, 610. 10.3389/fbioe.2020.00610 32850686 PMC7396513

[B71] Su H. Zhang X. He Y. Li L. Wang Y. Hong G. (2020). Transcriptomic analysis reveals the molecular adaptation of three major secondary metabolic pathways to multiple macronutrient starvation in tea (Camellia sinensis). Genes. 11 (3), 241. 10.3390/genes11030241 32106614 PMC7140895

[B72] Tomar S. Agarwal S. Singh H. Kumar R. Qureshi K. A. Jaremko M. (2023). Microalgae: a promising source for biofuel production. Biocatal. Agric. Biotechnol. 53, 102877. 10.1016/J.BCAB.2023.102877

[B73] Tsarpali M. Arora N. Kuhn J. N. Philippidis G. P. (2021). Lipid-extracted algae as a source of biomaterials for algae biorefineries. Algal Res. 57, 102354. 10.1016/j.algal.2021.102354

[B74] Ullah H. Gul B. Khan H. Akhtar N. Rehman K. U. Zeb U. (2022). Effect of growth medium nitrogen and phosphorus on nutritional composition of Lemna minor (an alternative fish and poultry feed). BMC Plant Biol. 22 (1), 214. 10.1186/s12870-022-03600-1 35468717 PMC9040223

[B75] Veronesi D. D’Imporzano G. Menin B. Salati S. Adani F. (2020). Organic wastes/by-products as alternative to CO2 for producing mixotrophic microalgae enhancing lipid production. Bioprocess Biosyst. Eng. 43 (10), 1911–1919. 10.1007/s00449-020-02381-x 32447512

[B76] Wang B. Rezenom Y. H. Cho K. C. Tran J. L. Lee D. G. Russell D. H. (2014). Cultivation of lipid-producing bacteria with lignocellulosic biomass: effects of inhibitory compounds of lignocellulosic hydrolysates. Bioresour. Technol. 161, 162–170. 10.1016/j.biortech.2014.02.133 24698742 PMC7702278

[B77] Wang Y. T. Zheng Q. P. Chai Y. Zhang Y. C. Zheng Y. Z. (2025a). Efficient pretreatment of tea stems using biocompatible ionic liquid and monoethanolamine: toward improved enzymatic hydrolysis and UV-blocking PVA-LNPs composite films. Chem. Eng. J. 523, 168378. 10.1016/j.cej.2025.168378

[B78] Wang Y. T. Chai Y. Zheng X. P. Du Y. P. Zhang Y. C. Zheng Y. Z. (2025b). Pretreatment of tea stem by biocompatible ionic liquids for enhanced enzymatic hydrolysis of cellulose and formation of UV-blocking chitosan film. Food Chem. 474, 143172. 10.1016/j.foodchem.2025.143172 39914347

[B79] Wu G. Chong J. W. R. Khoo K. S. Tang D. Y. Y. Show P. L. (2025). Upcycling food waste for microalgae cultivation toward lipid production in a closed-loop and system-integrated circular bioeconomy. In Biotechnol. Biofuels Bioprod., (Vol. 18. 74 10.1186/s13068-025-02679-6 40646635 PMC12255073

[B80] Xu J. Wei Y. Huang Y. Weng X. Wei X. (2023). Current understanding and future perspectives on the extraction, structures, and regulation of muscle function of tea pigments. Crit. Rev. Food Sci. Nutr. 63 (33), 11522–11544. 10.1080/10408398.2022.2093327 35770615

[B81] Yadav K. Vasistha S. Nawkarkar P. Kumar S. Rai M. P. (2022). Algal biorefinery culminating multiple value-added products: recent advances, emerging trends, opportunities, and challenges. 3 Biotech. 12 (10), 244. 10.1007/s13205-022-03288-y 36033914 PMC9402873

[B82] Zabochnicka M. Krzywonos M. Romanowska-Duda Z. Szufa S. Darkalt A. Mubashar M. (2022). Algal biomass utilization toward circular economy. Life 12 (10), 1480. 10.3390/life12101480 36294915 PMC9605372

[B83] Zhang S. Zhang L. Xu G. Li F. Li X. (2022). A review on biodiesel production from microalgae: influencing parameters and recent advanced technologies. Front. Microbiol. 13, 970028. 10.3389/fmicb.2022.970028 35966657 PMC9372408

[B84] Zhang Y. Lu Y. Pan D. Zhang Y. Zhang C. Lin Z. (2024). Efficient conversion of tea residue nutrients: screening and proliferation of edible fungi. Curr. Res. Food Sci. 9, 100907. 10.1016/j.crfs.2024.100907 39555019 PMC11565551

[B85] Zheng N. Lin X. Huang P. Liu Y. Bartlam M. Wang Y. (2023). Tea polyphenols inhibit blooms caused by eukaryotic and prokaryotic algae. Ecotoxicol. Environ. Saf. 265 (19), 115531. 10.1016/j.ecoenv.2023.115531 37778238

[B86] Zhou P. R. Zheng X. P. Du Y. P. Chai Y. Zhang Y. C. Zheng Y. Z. (2025). Effective pretreatment of tea stem *via* poly-deep eutectic solvent for promoting platform molecule production and obtaining fluorescent lignin. Int. J. Biol. Macromol. 297, 139922. 10.1016/j.ijbiomac.2025.139922 39824418

[B87] Zou L. G. Zheng D. L. Yao Y. T. Wen F. F. Li D. W. Yang Y. F. (2023). Polyphenols modulate microalgae metabolism with a particular increment in lipid accumulation. Fuel 352, 129085. 10.1016/j.fuel.2023.129085

